# Early evidence of a shift in juvenile fish communities in response to conditions in nursery areas

**DOI:** 10.1038/s41598-020-78181-w

**Published:** 2020-12-03

**Authors:** Sanja Matić-Skoko, Dario Vrdoljak, Hana Uvanović, Mišo Pavičić, Pero Tutman, Dubravka Bojanić Varezić

**Affiliations:** Institute of Oceanography and Fisheries, Meštrovićevo šetalište 63, P.O. Box. 500, 21000 Split, Croatia

**Keywords:** Environmental sciences, Marine biology, Biodiversity

## Abstract

A multivariate analysis of juvenile fish community data, sampled at two nursery sites at an interval of 17 years (2000—early, and 2017—late), was conducted to elucidate the trends of change in littoral juvenile fish communities along the eastern Adriatic coast. Fishing, trophic and taxonomic composition to the community data were analysed for possible causality. The ichthyofaunal composition differed significantly for Site, Period and all interactions. According to the mMDS ordination plot, four groups of communities were defined, with clear cyclicity. No patterns were found in species composition between sites in the early period, while the observed community changes were governed by the same pattern at both sites in the late period. The species that contributed most to the observed changes were non-commercial, small, benthic resident fishes, such as gobiids and blennids, or those associated with canopy alga for shelter and feeding. The analysis correctly allocated samples based on community information to Sites and Periods. The data obtained provided an invaluable opportunity to test for the generality of potential patterns of change in littoral fish communities, suggesting that significantly modified juvenile fish communities may be the result of constant human embankment and marine infrastructure construction along the coast in recent decades, rather than climate change or fishing pressure, as generally considered.

## Introduction

Mediterranean coastal waters are highly structured and fragile ecosystems. Encompassing a wide variety of bottom types, they have a remarkable diversity of littoral benthic communities at very small spatial scales that sustain high biodiversity and trophic complexity^1,2^. Estuaries and coastal areas are commonly acknowledged as highly productive and valuable ecosystems that provide a wide diversity of habitats for fish and that support fundamental ecological links with other environments^3^. Even for non-coastal fish, their role in early life stages, and as foraging and spawning grounds, has been highlighted. Nurseries overgrown by *Posidonia oceanica* seagrass meadows show spatial and temporal variations throughout the Mediterranean^4,5^, and are important habitats for adult and juvenile fishes^6,7^. However, the evidence on the importance of habitat characteristics in driving patterns of population dynamics is ambiguous^8^. As nurseries, coastal habitats are ecologically and economically important for the replenishment of coastal fish populations. Many fishes use shallow coastal habitats and estuaries to complete their life cycles, with larvae or early juveniles spending months to years in these environments before recruiting to coastal adult populations (see reviews by^3,9^). Scientific efforts to quantify habitat specific demographic rates are needed to better predict the effects of coastal habitats on the dynamics of exploited marine populations and sustainable exploitation rates^10^, and to determine connectivity among geographically segregated juveniles and adults. This is a key factor in the regulation of population dynamics, colonization patterns and resilience to harvest^9,11,12^.

Understanding the role of habitat use is particularly valuable from the conservation and management perspectives, since coastal habitats are increasingly vulnerable to anthropogenic stressors^13–18^. This can lead directly to habitat deterioration, causing loss of species and fisheries resources, reduced water quality and pollution. Marine ecosystems have been degraded to the extent that critical coastal habitats are no longer available or adequate to provide nursery, feeding, or reproductive functions, resulting in negative consequences on production and renewal of populations^19^. Many threats are known to weaken *Posidonia* habitats, such as invasive algae, eutrophication, trace metal accumulation, dredging, anchoring and increased turbidity^7^. Unfortunately, along the Mediterranean, including the Adriatic Sea, negative practices of marine construction in recent decades, such as embankment, gravel beaches nourishment, marinas, breakwaters and seawalls has been rampant, primarily to increase tourism facilities and capacities. Such artificial structures have the potential to modify marine physical conditions by interfering with water circulation and increasing sedimentation and pollution, leading to biological homogenization^20^. However, they can also increase the availability of hard substrata for larval recruitment, resulting in the development of a diverse sessile community and associated fish fauna^21^. A change in community species composition may also be the consequence of migration of different fish species according to their respective dispersal strategies.

Without a doubt, climate change has driven significant changes in marine ecosystems in recent decades, particularly in coastal areas facing compositional and structural changes. Specifically, alterations in species distributions and ranges, composition of species assemblages, and biodiversity have all been convincingly linked to rising temperatures^22,23^. A recent meta-analysis indicated that the geographic distributions of marine species are shifting toward the poles much faster and occupying their potential latitudinal ranges to a greater extent than their terrestrial counterparts^24^. On the other side, the impacts induced by fishing in marine ecosystems are relatively well known and wide-ranging, as both direct (abundance decrease, changes in size and species composition, modifications of population parameters) and indirect effects (trophic shift, bottom disturbance) acting on short- and long-term temporal scales^25,26^. Although anthropogenic impacts may be responsible for changes in coastal communities, most experts still agree that fisheries mismanagement can generate the most far-reaching consequences^25,27^.

The ability to understand how human activities, environmental factors and ecological components interact and influence each other is of growing importance^28^. However, establishing causal relationships between a wide range of stressors and effects on marine ecosystems, at the individual, species or community level, is a difficult task that requires the use of multiple lines of evidence, particularly in the Mediterranean region^29^. The Adriatic Sea is considered the most exploited basin of the entire Mediterranean Sea^30^. Historically, it has been subjected to vast anthropogenic pressures and is characterized by a wide spectrum of environmental variability^31^. In this study, selected sites are recognized as essential nursery areas^32–34^ but over last 2 decades we observed general decrease of seagrass^35^ and continuous human interventions in term of different harbour constructions associated with these sites. We conduct multivariate analysis of juvenile fish community data, sampled in an early (2000–2001) and late period (2017–2018) specifically planned to provide a consistent time series to elucidate the shift in littoral juvenile fish communities along the eastern Adriatic coast. Changes in fish community abundance, diversity and structure were examined, encompassing both temporal and spatial replication. This analysis provided an invaluable opportunity to test for the generality of potential patterns of change in littoral fish communities along the eastern Adriatic coast. Potential causes of those patterns were addressed, and we identified the most vulnerable fish species and species that can benefit from the situation if the observed trend of changes continues.

## Results

### Community indices

A total of 144 small beach seine samples were taken in both periods at both sites, and Table 1 lists all captured species with their corresponding rank (Table 1). A total of 37,056 fish individuals belonging to 22 families and 87 species were caught in this survey. The most frequently observed species were *Atherina boyeri*, *Pomatoschistus marmoratus*, *Sarpa salpa* and *Symphodus cinereus,* which were present in more than 80% of hauls, while most species (91.7%) were present in less than 50% of hauls. Although the total number of samples per period and site was the same (36), the total number of species decreased by 22% and 15.4% for Duće and Sovlja, respectively, from the early to the later period, though this decline was only significant at the Duće site (p < 0.05; Table 2). However, the observed changes in the total number of individuals per period was more dramatic and the change was statistically significant at both sites (Table 2). Namely, the mean overall density of fish at the Duće site in 2000 was 204.53 fish/haul, in comparison to 90.72 fish/haul in 2017 (decline of 44.3%), while the mean overall density of fish was 459.44 fish/haul and 274.64 fish/haul, respectively (decline of 59.8%) for the early and late period at the Sovlja Site.

**Table 1:** List of all fish species recorded in the early (2000–2001) and late (2017–2018) period at both sites (Duće and Sovlja) in alphabetical order with their occurrence frequencies (%) and rank.

Species	Family	Trophic category	Fishing value	DUĆE				SOVLJA			
				2000		2018		2000		2018	
				%	Rank	%	Rank	%	Rank	%	Rank
*Aidablennius sphynx*	Blennidae	BEN	NC					< 0.1			
*Arnoglossus kessleri*	Bothidae	MECA 2	NC					< 0.1			
*Arnoglossus laterna*	Bothidae	MECA 2	NC	0.53	15	2.02	5	0.14	26	< 0.1	
*Arnoglossus thori*	Bothidae	MECA 2	NC					< 0.1			
*Atherina boyeri*	Atherinidae	MICA	MC	20.28	1	73.91	1	23.16	2	51.78	1
*Atherina hepsetus*	Atherinidae	MICA	MC	12.90	4	1.01	9	26.06	1	1.66	9
*Belone belone*	Belonidae	PLA	MC	< 0.1		< 0.1		< 0.1			
*Boops boops*	Sparidae	MICA	MC							0.16	23
*Bothus podas*	Bothidae	MECA 2	NC					< 0.1		< 0.1	
*Callionymus maculatus*	Callionymidae	BEN	NC					< 0.1			
*Callionymus pusillus*	Callionymidae	BEN	NC	0.42	18	1.01	8	0.18	25		
*Callionymus risso*	Callionymidae	BEN	NC	0.98	12	1.84	6	< 0.1		< 0.1	
*Chelon labrosus*	Mugilidae	POM	MC	0.16	23	< 0.1		1.14	15		
*Coris julis*	Labridae	MECA 1	NC			< 0.1		< 0.1		< 0.1	
*Deltentosteus collonianus*	Gobidae	BEN	NC							< 0.1	
*Dentex dentex*	Sparidae	MECA 2	HC			< 0.1		< 0.1		< 0.1	
*Diplodus annularis*	Sparidae	MECA 2	MC	3.53	6	1.29	7	1.20	14	1.92	7
*Diplodus puntazzo*	Sparidae	MECA 2	HC	1.36	11			0.82	16	0.43	17
*Diplodus sargus*	Sparidae	MECA 2	HC	< 0.1				< 0.1		< 0.1	
*Diplodus vulgaris*	Sparidae	MECA 2	HC	1.98	9	0.49	10	0.54	19	1.01	12
*Echiichthys vipera*	Trachinidae	MECA 2	NC	0.46	17	0.49	11	< 0.1			
*Gobius bucchichi*	Gobidae	BEN	NC					0.20	23		
*Gobius cobitis*	Gobidae	BEN	NC	0.12	26			0.18	24		
*Gobius couchi*	Gobidae	BEN	NC			< 0.1		0.11	29	1.91	8
*Gobius cruentatus*	Gobidae	BEN	NC							< 0.1	
*Gobius fallax*	Gobidae	BEN	NC					< 0.1		0.93	13
*Gobius geniporus*	Gobidae	BEN	NC	0.14	25			< 0.1			
*Gobius niger*	Gobidae	BEN	NC	< 0.1		< 0.1		1.85	9	2.08	6
*Hippocampus guttulatus*	Syngnathidae	PLA	NC	< 0.1							
*Knipowitschia panizzae*	Gobiidae	BEN	NC	< 0.1							
*Labrus viridis*	Labridae	MECA 1	NC	< 0.1							
*Lepidotrigla cavillone*	Triglidae	MACA	NC	< 0.1							
*Lichia amia*	Carangidae	MACA	MC	< 0.1				0.11	28		
*Lipophrys dalmatinus*	Blennidae	BEN	NC					< 0.1			
*Lipophrys pavo*	Blennidae	BEN	NC	< 0.1				0.32	21		
*Lithognathus mormyrus*	Sparidae	MECA 2	HC	14.64	3	< 0.1		1.24	13	< 0.1	
*Liza aurata*	Mugilidae	POM	HC	0.64	13	0.34	13	13.11	3	< 0.1	
*Liza ramada*	Mugilidae	POM	HC	0.16	24	0.46	12	2.13	7	< 0.1	
*Liza saliens*	Mugilidae	POM	MC	< 0.1		0.15	15	1.28	12	0.17	22
*Monochirus hispidus*	Soleidae	MECA 2	NC			< 0.1					
*Mugil cephalus*	Mugilidae	POM	HC	< 0.1				0.59	18		
*Mullus barbatus*	Mullidae	MECA 2	HC			5.76	2	7.71	4	1.52	10
*Mullus surmuletus*	Mullidae	MECA 2	HC	1.40	10	< 0.1		1.97	8	< 0.1	
*Nerophis maculatus*	Syngnathidae	PLA	NC	< 0.1		< 0.1					
*Nerophis ophidion*	Syngnathidae	PLA	NC	2.85	8	0.09	18	< 0.1		< 0.1	
*Oblada melanura*	Sparidae	PLA	HC					< 0.1		< 0.1	
*Odondebuenia balearica*	Gobidae	BEN	NC							< 0.1	
*Oedalechilus labeo*	Mugilidae	POM	NC					0.48	20		
*Ophidion rochei*	Ophidiidae	PLA	NC	< 0.1							
*Pagellus acarne*	Sparidae	MECA 2	MC			< 0.1				3.53	4
*Pagellus erythrinus*	Sparidae	MECA 2	HC	< 0.1		< 0.1		< 0.1		0.22	20
*Pagrus pagrus*	Sparidae	MECA 2	HC							< 0.1	
*Parablennius gattorugine*	Blennidae	BEN	NC							< 0.1	
*Parablennius incognitus*	Blenniidae	BEN	NC	< 0.1							
*Parablennius sanguinolentus*	Blenniidae	BEN	NC	0.38	19			0.10	30	< 0.1	
*Parablennius tentacularis*	Blenniidae	BEN	NC	< 0.1				0.13	27	0.68	14
*Pomatoschistus bathi*	Gobidae	BEN	NC					< 0.1		0.35	18
*Pomatoschistus canestrinii*	Gobiidae	BEN	NC	0.50	16						
*Pomatoschistus marmoratus*	Gobiidae	BEN	NC	18.02	2	4.59	3	4.33	6	< 0.1	
*Pseudaphya ferreri*	Gobidae	BEN	NC					< 0.1		< 0.1	
*Sardina pilchardus*	Clupeidae	PLA	HC	11.78	5	3.70	4	< 0.1		< 0.1	
*Sarpa salpa*	Sparidae	HER	HC	3.10	7	0.28	14	6.12	5	18.71	2
*Scophthalmus maximus*	Scopthalmidae	MECA 2	MC					< 0.1			
*Scorpaena porcus*	Scorpaenidae	MACA	MC	< 0.1		< 0.1		< 0.1		0.64	15
*Serranus hepatus*	Serranide	MACA	MC					< 0.1		0.52	16
*Serranus scriba*	Serranide	MECA 1	MC			< 0.1				0.19	21
*Solea kleini*	Soleidae	MECA 2	MC	< 0.1							
*Solea solea*	Soleidae	MECA 2	HC	< 0.1		< 0.1		< 0.1			
*Sparus aurata*	Sparidae	MECA 3	HC	< 0.1				0.65	17		
*Sphyraena sphyraena*	Sphyraenidae	MACA	MC					< 0.1		< 0.1	
*Spicara smaris*	Centracanthidae	MICA	HC					< 0.1		0.14	24
*Spondyliosoma cantharus*	Sparidae	MECA 2	HC							< 0.1	
*Symphodus cinereus*	Labridae	MECA 1	NC	0.35	20	1.53	7	1.31	11	6.29	3
*Symphodus ocellatus*	Labridae	MECA 1	NC	1.29	12	< 0.1		1.48	10	2.80	5
*Symphodus roissali*	Labridae	MECA 1	NC	0.16	22			< 0.1			
*Symphodus rostratus*	Labridae	MECA 1	NC	< 0.1							
*Symphodus tinca*	Labridae	MECA 1	MC			< 0.1		< 0.1		1.22	11
*Syngnathus abaster*	Syngnathidae	PLA	NC	0.11	27			< 0.1			
*Syngnathus acus*	Syngnathidae	PLA	NC	0.19	21	< 0.1		< 0.1		< 0.1	
*Syngnathus tenuirostris*	Syngnathidae	PLA	NC			< 0.1		< 0.1		0.28	19
*Syngnathus typhle*	Syngnathidae	PLA	NC	0.61	14	0.15	16	< 0.1		0.10	25
*Trachinus draco*	Trachinidae	MECA 2	MC							< 0.1	
*Trigla lucerna*	Triglidae	MECA 2	HC	< 0.1		0.12	17	< 0.1		< 0.1	
*Trigla lyra*	Triglidae	MECA 2	MC			< 0.1					
*Tripterygion tripteronotum*	Tripterygiidae	BEN	NC					< 0.1		< 0.1	
*Zeugopterus regius*	Gobidae	BEN	NC					< 0.1			
*Zosterisessor ophiocephalus*	Gobidae	BEN	NC					0.31	22	< 0.1	
Number of samples				36		36		36		36	
Number of species				50		39		65		55	
Number of individuals				7363		3266		16,540		9887	
Mean number of fish/haul				204.53		90.72		459.44		274.64	
Mean number of species/haul				1.39		1.08		1.81		1.53

**Table 2:** Univariate PERMANOVA analysis results of abundance, diversity indices, fishing category and ecological category of juvenile fish communities from both sites (Duće and Sovlja) and periods (early 2000 and late 2017) in the Adriatic Sea.

	Factor	DUĆE		SOVLJA	
		Pseudo-F	P (perm)	Pseudo-F	P (perm)
Diversity indices	S	42.1	**0.0001**	2.397	0.115
	N	10.984	**0.0003**	10.495	**0.002**
	D	29.587	**0.0001**	0.092	0.823
	J'	4.022	**0.043**	0.563	0.466
	SW	5.43	**0.019**	0.237	0.653
	D	12.323	**0.0004**	0.07	0.851
	Δ*	2.357	0.128	1.076	0.303
Fishing indices^a^	NC	42.342	**0.0001**	2.495	0.109
	HC			20.274	**0.0001**
Trophic indices^a^	BEN	5.389	**0.024**	1.213	0.266
	MECA2	15.376	**0.001**	3.879	0.056

Bold values indicate the significant.

^a^Undefined resemblances between samples for MC, HC for Duće and other trophic categories for both sites.

Although the most abundant species caught at both sites during the study period were also ranked high by abundance in both the early and late period at both sites, the ranking by abundance and relative contribution of species to the total catch differed markedly between periods (Table 1). However, there were some interesting exceptions. For example, *S. salpa* ranked seventh at Duće in the early period but was not within in the top ten in the late period due to its contribution to abundance of less than 0.3%. Inversely, at the Sovlja site, *S. salpa* ranked fifth in the early period compared with second and a contribution of 18.7% in the late period. However, the most obvious example of change was the decline in abundance of *Lithognathus mormyrus* at Duće between the early and late period. This mezocarnivorous species was ranked third at Duće (14.6%) in the early period, though its abundance dropped to less than 0.1% in the late period. It is worthwhile mentioning the small gobiid species, *P. marmoratus*, as one of the most dominant and frequent species during the study period. In the early period, *P. marmoratus* was ranked second and sixth at Duće and Sovlja, respectively, while in late period its abundance decreased at Duće (to third) while at Sovlja it all but disappeared (< 0.1% in the total sample).

**Figure 1:**
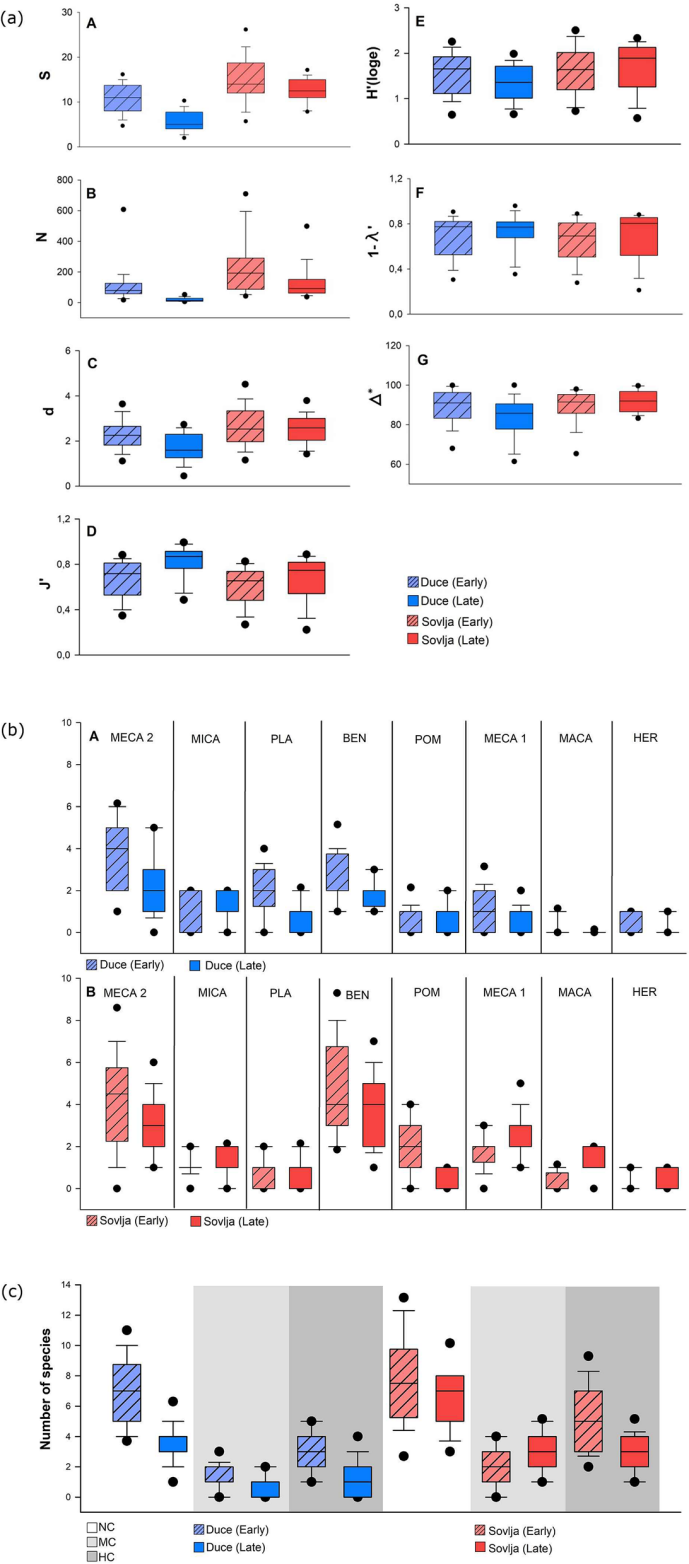
Box plots of median (± standard deviation) for (**a**) diversity indices; (**b**) fishing categories and (**c**) trophic categories for juvenile fish abundances from both sites (Duće and Sovlja) and both periods (early 2000 and late 2017) in the Adriatic Sea.

Of the diversity indices analysed, the same significantly negative pattern was observed for Margalef’s species richness (d), Shannon–Wiener diversity and Simpson’s indices (1-ƛ) for the Duće site. However, these diversity indices for the Sovlja site showed variability between periods but with no obvious temporal shift (Table 2, Fig. 1a). Pielou evenness showed a significant (p < 0.05) positive trend at the Duće site. The decreasing trend of diversity indices was the result of the lower number of species caught at both sites in 2017. However, this decrease was not seen among those fish species occurring only once or with one individual in 2000, i.e. that they were not observed in 2017. Instead, it was the highly ubiquitous and abundant fish species present in the early period that disappeared later in the study, like *L. mormyrus* or *Mullus surmuletus*.

In terms of ecological categories related to trophic level, the number of small benthic fishes (BEN) sampled in Duće and Sovlja decreased from 12 to 5 and from 20 to 15, respectively between periods (Fig. 1b), though this decrease was only significantly different for Duće (p < 0.05, Table 2). Although fewer species of MECA2 were caught in the late period at both sites, this decrease was significant only for Duće and at the limit of significance for Sovlja. The number of marine microcarnivores (MICA), mezocarnivores Labridae (MECA1), herbivores (HERB), omnivores (OMN) and planktivores (PLA) were the same or very similar in both periods at both sites (Tables 1, 2, Fig. 1b).

Although, the number of non-commercial fishes (NC) sampled in Duće and Sovlja decreased from 27 to 16 and from 35 to 24, respectively (Fig. 1c), that change was significantly different between periods only for the Duće site (p < 0.05, Table 2). For high-commercial fishes (HC), decreases were observed at both sites, though the difference was statistically significant only at the Sovlja site.

There were no significant differences in temperature (p > 0.05) between sites and periods however the differences were more pronounced between periods for each site than between sites for the early and late periods. However, significant differences in salinity (p = 0.001) were obtained between sites and periods over the study period.

### Multi-parameter comparison

PERMANOVA analysis of the ichthyofaunal compositions at the Duće and Sovlja sites, derived from seine samples in the early and late periods, found significant differences for Site, Period, and Months (Table 3). All interactions were also significant for both sites and periods.

**Table 3:** Summary of PERMANOVA results for the multivariate analysis of overall matrices constructed from juvenile fish abundances from both sites (Duće and Sovlja) and both periods (early 2000 and late 2017) in the Adriatic Sea.

	df	MS	Pseudo-F	P
Site (Si)	1	85,448	70.742	0.0001
Period (Pe)	1	38,532	31.901	0.0001
Month (Mo)	11	6446.7	5.337	0.0001
**Interactions**				
Si × Pe	1	30,359	25.134	0.0001
Si × Mo	11	3588	2.97	0.0001
Pe × Mo	11	4272.8	3.537	0.0001
Si × Pe × Mo	11	3874.3	3.208	0.0001
**Residuals**	96	1207.9		
Total	143			

**Figure 2:**
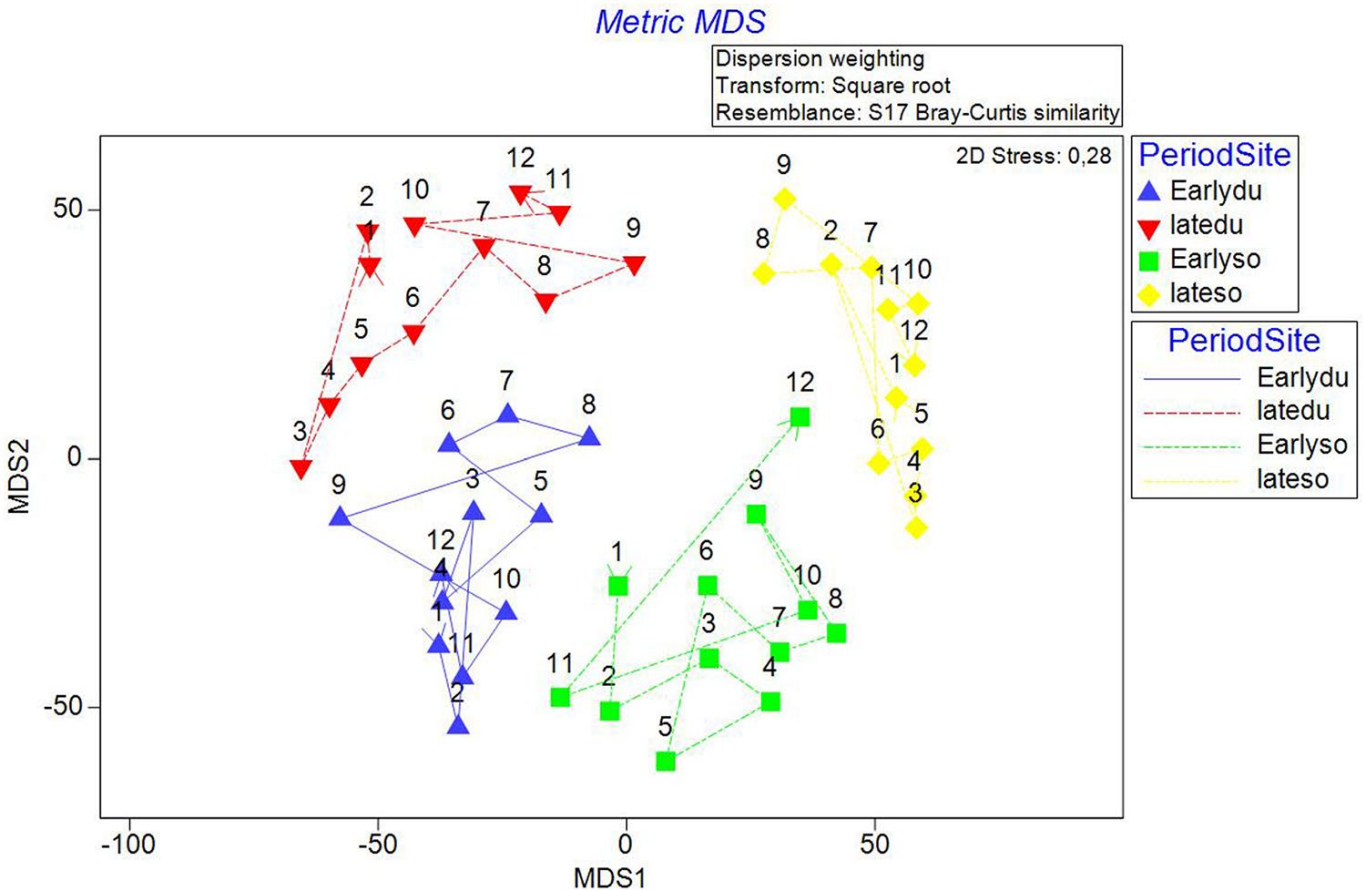
Metric MDS for juvenile fish abundances for PeriodSite (sites Duće and Sovlja and periods early 2000 and late 2017) in the Adriatic Sea with marked cyclicity for all four groups.

On the metric MDS ordination plot, derived from the distance among centroids matrices, four groups were defined, clearly separating both period and site. The early period data were positioned towards the bottom of the figure and the late period data towards the top, while for site, Duće was positioned towards the left and Sovlja towards the right (Fig. 2).

There would appear to be some cyclicity in the sample relationships, with matching distances between points placed equidistantly around a circle. The RELATE Model automatically constructs a simple cyclicity that could be the consequence of a seasonal signal, with the assemblage structure for later months gradually returning to the start. It seems that in early period there is no similar pattern between the Duće and Sovlja sites (Rho = 0.098, p = 0.240). Interesting, there is also no similar pattern between early and late period at the Sovlja site (Rho = 0.222, p = 0.07), though the species composition in late period at both sites is governed by the same pattern (Rho = 0.565, p < 0.05). Two-way crossed ANOSIM tests demonstrated and confirmed that the fish fauna composition was significantly related to both Sites (R = 0.659, p = 0.001) and Periods (R = 0.485, p = 0.001). Moreover, one-way ANOSIM for the interaction PeriodSite confirmed that the magnitude of difference was lowest between sites in the early period (R = 0.419; p = 0.001) and highest in the late period (R = 0.899; p = 0.001).

The SIMPER routine revealed that ten species (Table 4) in varying order of percentage contribution are responsible for the most (> 50%) dissimilarities in the interaction PeriodSite. It was evident that the species giving the highest contribution to the Duće Site (*P. marmoratus* and *Nerophis ophidion*) in the early period were replaced with other species, while at the Sovlja site, the top two species contributing to cumulative dissimilarity (*S. cinereus* and *Gobius niger*) increased their abundance while *S. salpa* and *Symphodus ocellatus* decreased in total number. Since these species are all non-commercial (NC), such as the small, benthic resident fish like *P. marmratus* and *G. niger* or species associated with algae for shelter and feeding (*S. salpa*, *Symphodus* sp.), changes in their abundance may be related to changes in sediment composition and vegetation cover.

**Table 4:** SIMPER: species contributing most to the dissimilarity, in terms of abundance of juvenile fish abundances from both sites (Duće and Sovlja) and both periods (early 2000 and late 2017) in the Adriatic Sea.

Species	Early Duće		Late Duće		Early Sovlja		Late Sovlja	
	Cont. (%)	Cumul. (%)	Cont. (%)	Cumul. (%)	Cont. (%)	Cumul. (%)	Cont. (%)	Cumul. (%)
*Pomatoschistus marmoratus*	28.91	28.91			10.65	21.83		
*Nerophis ophidion*	12.12	41.07						
*Sygnathus typhle*	9.64	50.71						
*Arnoglossus laterna*			31.91	31.91				
*Callionymus risso*			24.76	56.67				
*Symphodus cinereus*					11.18	11.18	25.67	25.67
*Gobius niger*					9.73	31.56	10.22	48.28
*Sarpa salpa*					9.07	40.64		
*Symphodus ocellatus*					7.73	78.37		
*Scorpaena porcus*							12.39	38.06

Cont. = contribution of species, Cumul = cumulative contribution.

**Figure 3:**
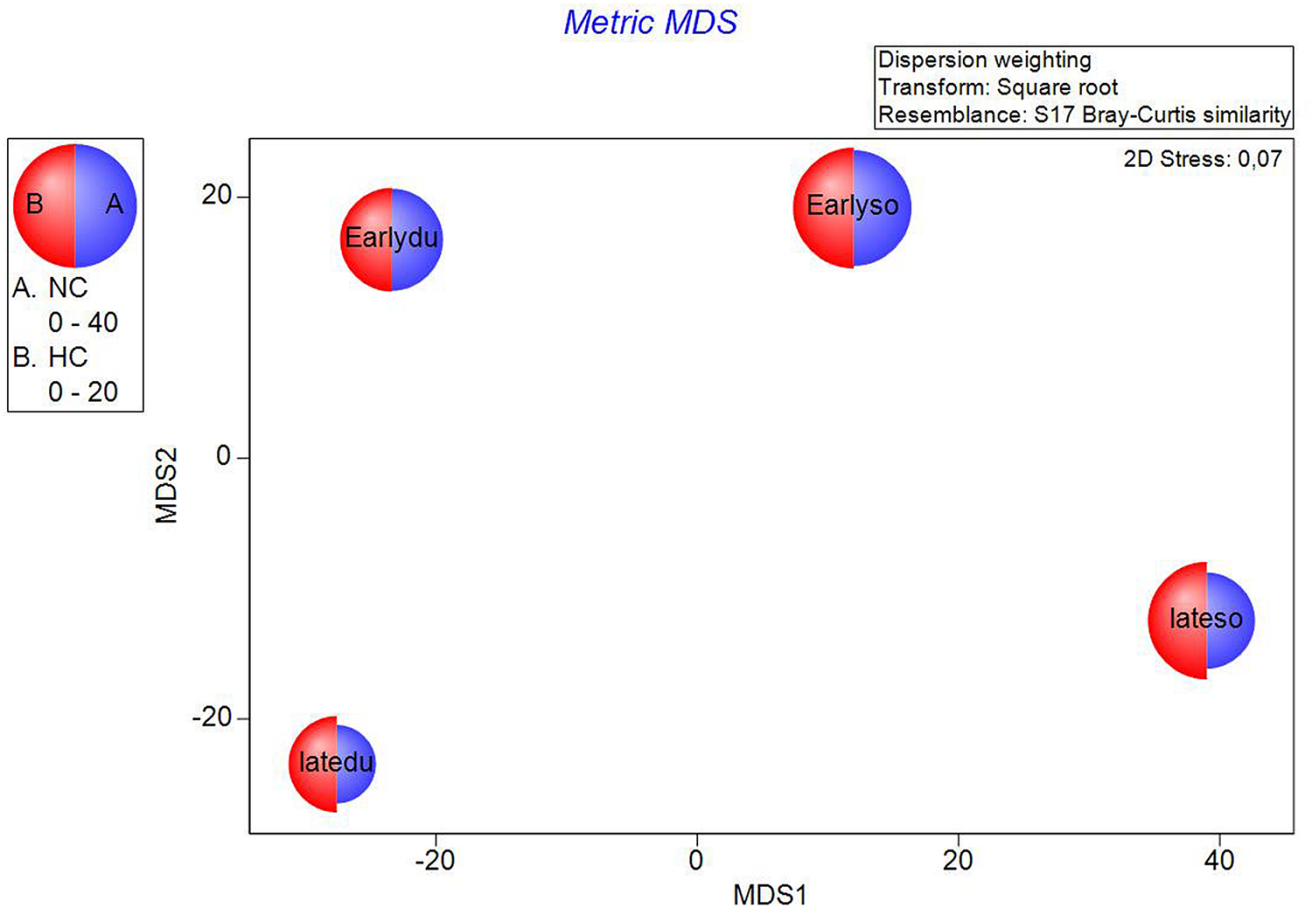
The metric MDS of juvenile fish abundance data performed on transformed fishing categories values showing the patterns across the four groups of interest. Each half-circle corresponds to NC (non-commercial) and HC (high commercial) and its size reflects the contribution of each category composition to the obtained distance.

**Figure 4:**
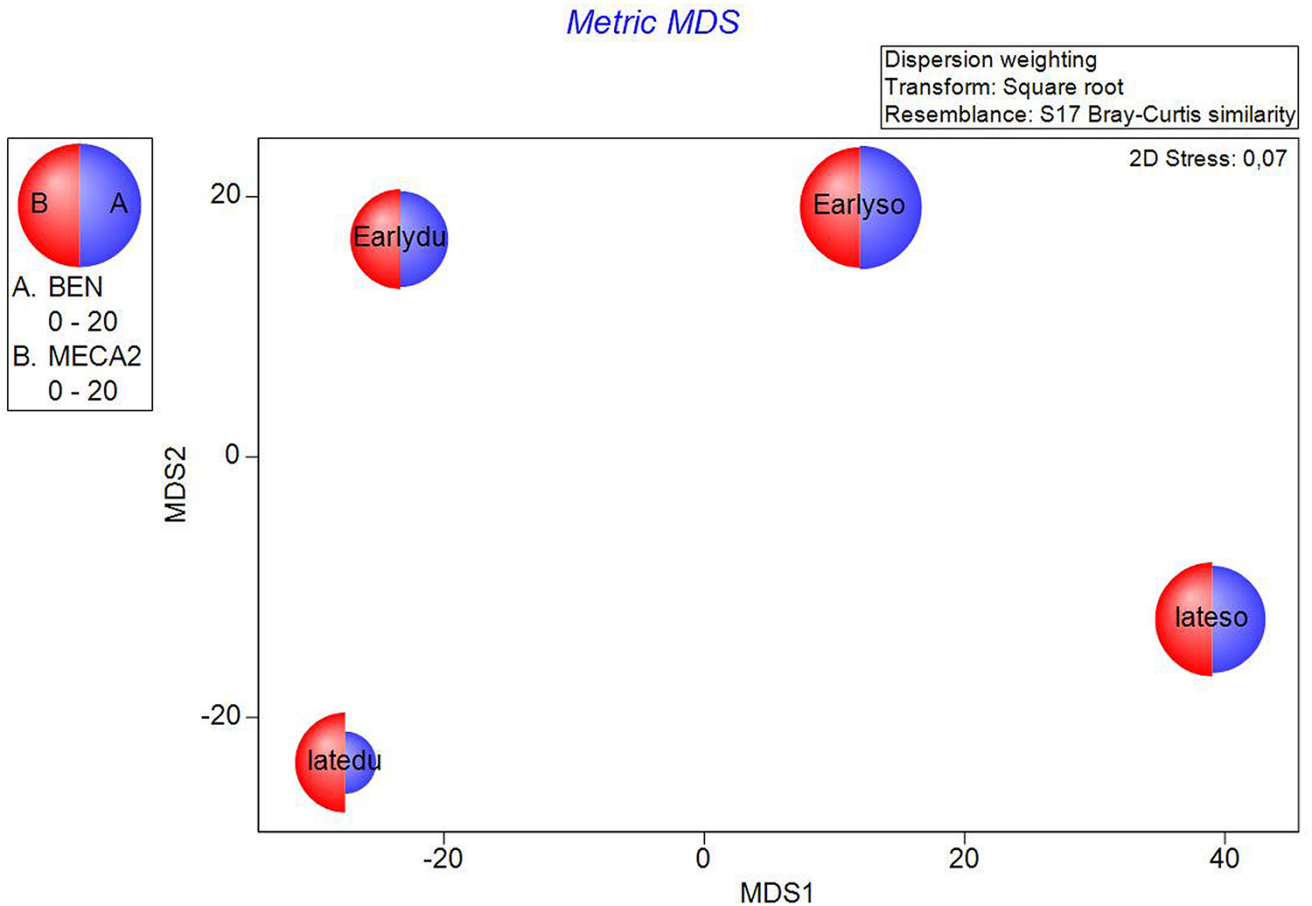
The metric MDS of juvenile fish abundance data performed on transformed trophic category values showing the patterns across the four groups of interest. Each half-circle corresponds to BEN (benthic fish) and MECA2 (mezocarnivorus fish) and its size reflects the contribution of each category composition to the obtained distance.

**Figure 5:**
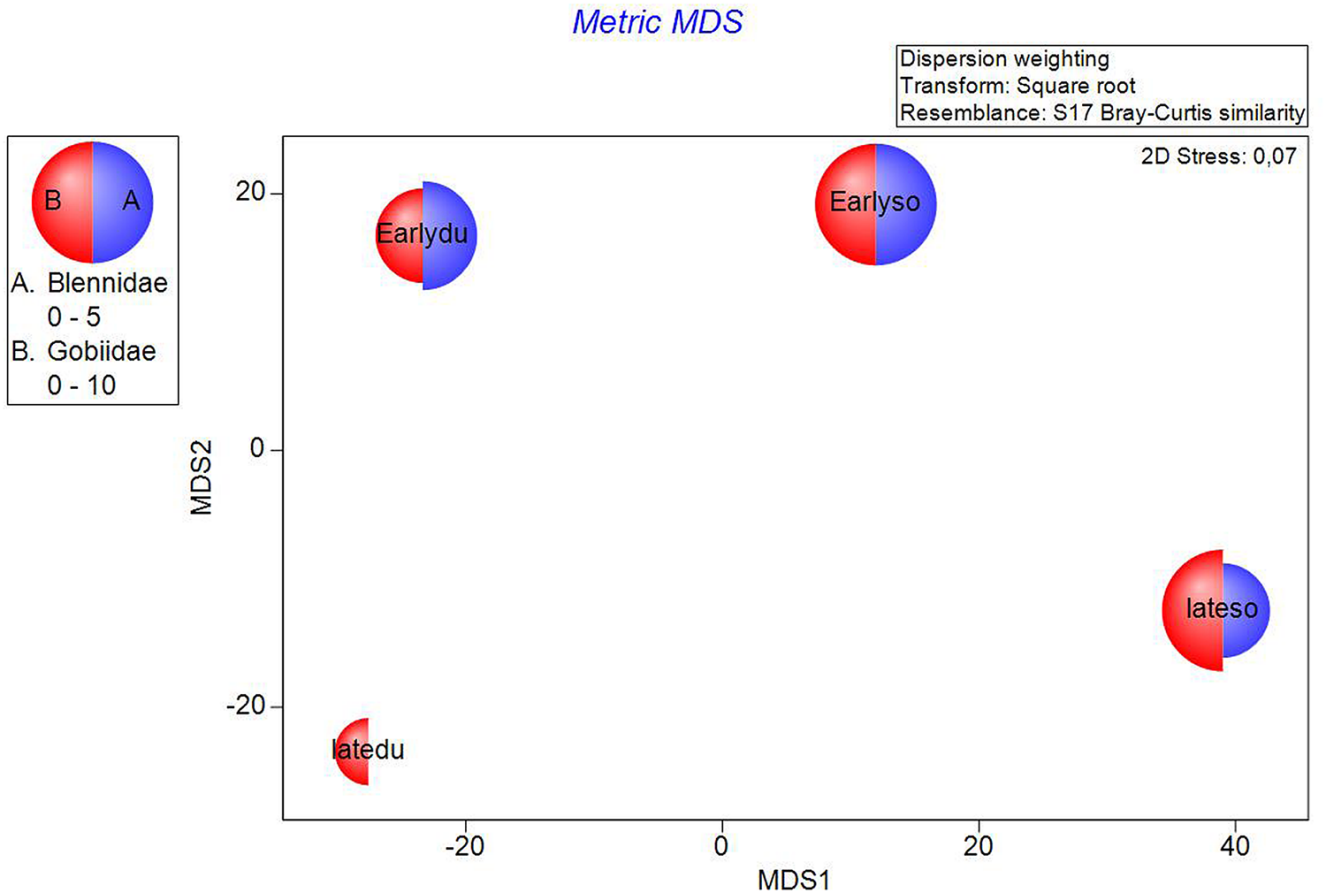
The metric MDS of juvenile fish abundance data performed on transformed taxonomic categories values showing the patterns across the four groups of interest. Each half-circle corresponds to the families Gobiidae and Blennidae and its size reflects the contribution of each category composition to the obtained distance.

We related fishing value (Fig. 3), trophic value (Fig. 4) and taxonomic composition (Fig. 5) directly to the juvenile community on the mMDS ordination plot. Those values for the four groups of factors (PeriodSite) were plotted as segmented bubbles of proportional sizes. They showed a clear separation of the early and late period with higher similarity between sites in the early period (closer position). In the late period, the percentage of non-commercial species decreased at both sites, particularly at the Duće site (Fig. 3). That is further confirmed by the fact that Gobiidae fully disappeared from Duće while Blennidae (both NC families) decreased at both sites (Fig. 4). Moreover, Fig. 5. showed that the percentage of the whole BEN group, representing small benthic fishes, decreased at both sites, though this decline was more evident at the Duće site, particularly in comparison with MECA 2 (mostly fishes the families Sparidae and Mullidae that feed on small crabs and polychaetes).

**Figure 6:**
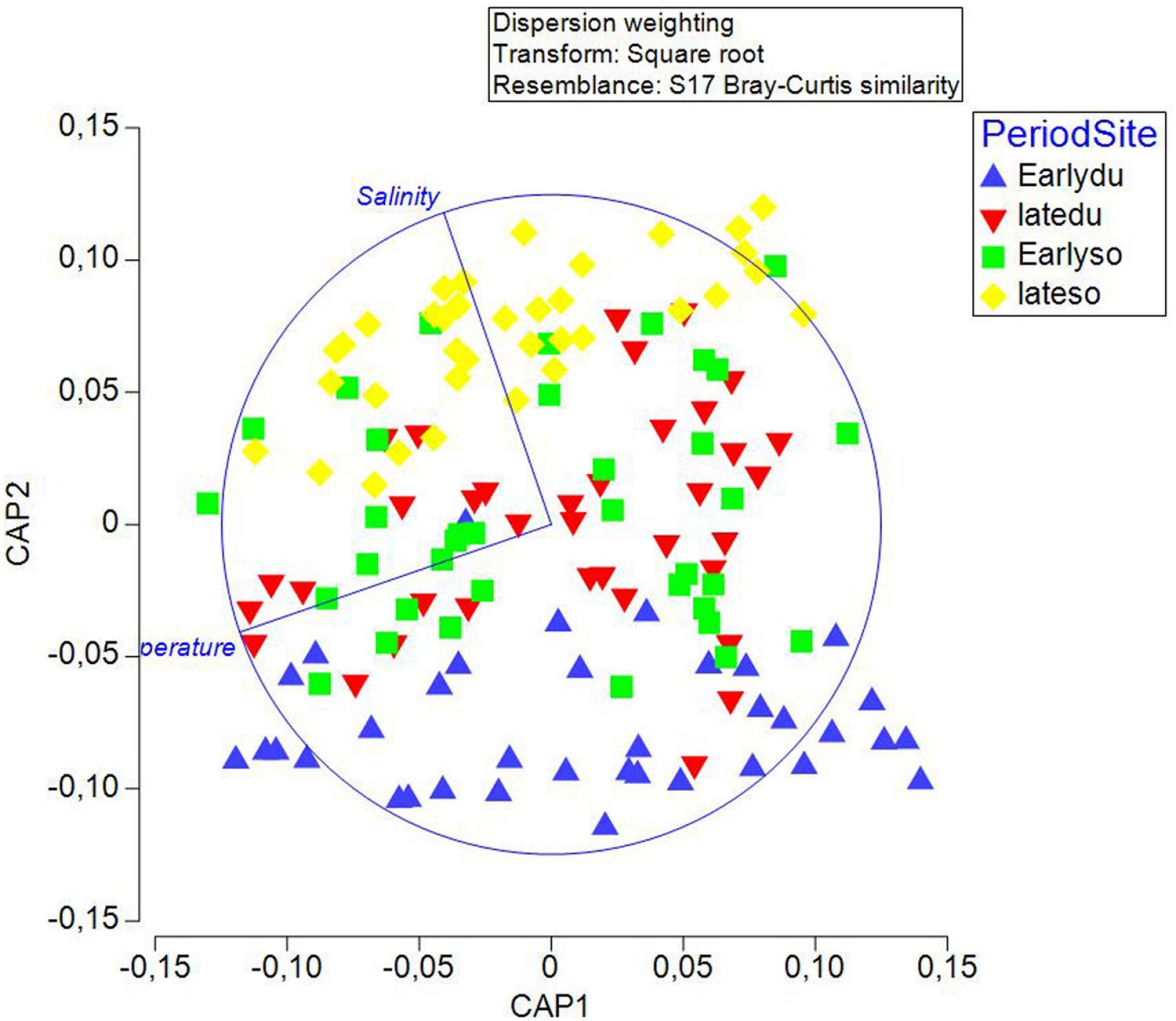
Canonical variate plot (CAP) of the juvenile fish abundance data on the environmental (continuous quantitative) values of temperature and salinity. The distance matrix based on community abundance data was related to the distance matrix based on environmental data.

In addition, we ran a CAP analysis and related the environmental data to the community composition information. Temperature and salinity values for the four groups of factors (Periods × Site) were plotted as distances among centroids based on community data (Fig. 6), revealing that temperature had no or little influence on community composition, while salinity had a positive effect on the late period at Sovlja (mean salinity was 36.6 ppm and 38.5 ppm for the early and late periods at Sovlja, respectively).

**Figure 7:**
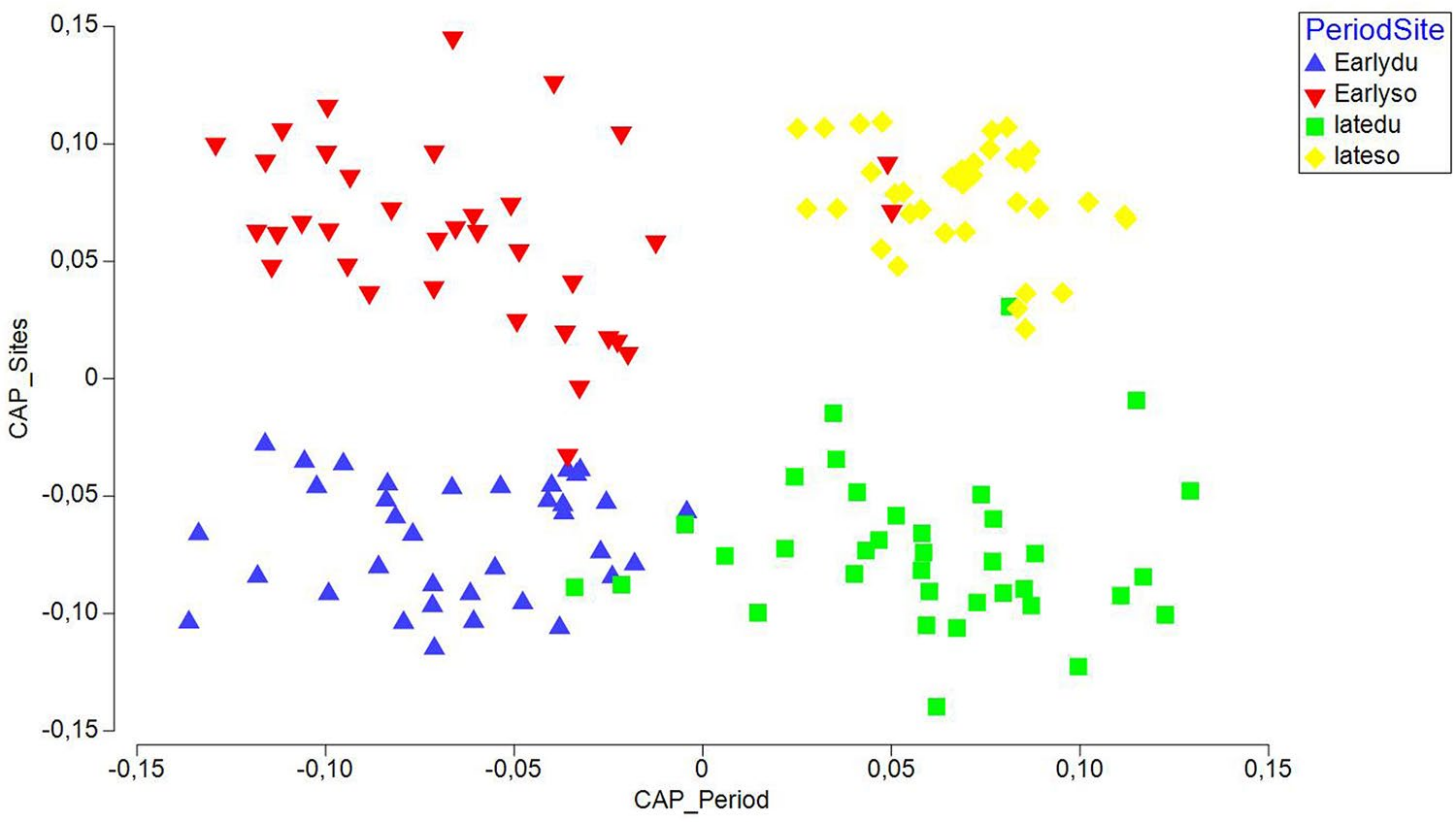
Canonical variate plot (CAP) for juvenile fish abundances sampled in the early (2000) and late (2017) period at both sites (Duće and Sovlja) in the Adriatic Sea, grouped by “Period” and “Species”.

Finally, a separate CAP analysis was run for each of the two factors (“Site” and “Period”) and this also gave successful discrimination for site and period. In particular, 97.2% of early period samples were correctly allocated based on the community composition information, and 95.8% samples to the late period. The same was observed for sites with 98.6% and 97.2% samples, which were correctly allocated to site. The two-way CAP plot obtained by merging the output scores for the CAP analysis of “Site” and “Period” showed a rather clear separation of the four groups (Fig. 7). It is apparent that the early samples for both sites are positioned to the left and the late samples are positioned on the right side of graph. In contrast, Duće samples were consistently clustered downwards, and Sovlja samples upwards.

## Discussion

This study describes multivariate analysis of community data together with environmental, fishing and trophic level indices, aimed at elucidating the main reason for the observed negative decadal changes in juvenile fish community composition in two nursery areas.

### Community indices

Most dramatic changes were observed in the number of individuals at both sites (decline of more than 44%). Generally, such a situation could result from a range of environmental and anthropogenic pressures, particularly climate change and overfishing^18,29,36^. High fishing pressures also lead to ecological changes, decreasing fish stocks and modifying their demographic structure^25,26,37^. This can impair the capacity of fish resources to grow and renew themselves^38^. Reductions in spawning-stock biomass and subsequent recruitment and fishery production in many exploited marine and estuarine populations have increased concerns over the multiple effects of fishing, habitat loss and degradation on the resilience and persistence of exploited marine fisheries populations^8,29,39^. The decrease in abundance at the Duće site was smaller than at the Sovlja site, which could be due to the fact that this estuarine area is more productive^19^, and has a greater potential to abate disturbances and preserved the community. In addition, just three species (*S. salpa*, *L. morymrus* and *P. marmoratus*) were highlighted as having the most evident negative temporal trend in abundance from the early to late period at the Duće site. Warm-water, herbivorous *S. salpa* is dependent on canopy-forming alga, which are mostly cold-water seaweed and ecological engineers throughout the Mediterranean Sea^40,41^. However, the analysis revealed that temperature and salinity had little to no influence on community composition at the Duće site, and thus it is more likely that species associated with algae for shelter and feeding, like *S. salpa,* experienced a decrease in their abundance due to changes in sediment composition and consequently vegetation cover. Changes in sediment or bottom type can be relate to different material accumulation following the alteration of hydrographic conditions stemming from embankment or gravel beaches nourishment and marine infrastructure building. Also, such accumulation can prevent continuous supply of fresh water throughout the year and thus could be responsible for the established statistically significant difference in salinity between the early and late period. Also, a sudden and drastic decline of *C. nodosa* meadow occurs due to increased seawater turbidity that resulted from increased terrigenous input, resuspension of sediment and elevated autotrophic biomass^35^.

Current coastal development locally may deplete many native marine species, while offering limited possibilities for their expansion. Multiple disturbances are affecting canopy-forming algae globally^40^. Also, the structural complexity of rocky sub-littoral zones influences where fish species feed and take shelter. In this area, herbivorous and omnivorous fishes seem to control both algae and invertebrates^42^. Usually, *L. mormyrus* is associated with soft bottoms by its ecological traits, and it has exhibited high mortality rates and a high percentage of the catch under the minimum legal size in the recreation fishery^43^. Also, crustacean ectoparasites pose threats to *L. mormyrus* and influence its abundance^44^. *P. marmoratus* inhabits coastal lagoons as semi-isolated ecosystems exposed to wide fluctuations of environmental conditions and subject to habitat fragmentation, thus environmental variables and habitat discontinuity may shape the presence and abundance of this species^45^.

Interestingly, a significant decrease in the number of species was observed at the Duće site, but not at Sovlja. We hypothesised that this change is more likely related to bottom and hydrographic changes than environmental or fishing pressures. The Duće site is located about 900 m from the river Cetina mouth, and under the certain influence of fresh water inflows^33,34^. On the other hand, this area has been subjected to pronounced infrastructural intervention over the last 20 years (breakwater construction at the mouth, formation of sandy beaches by constant nourishment, sand extraction)^46^. Sovlja Cove is situated near Šibenik and is a typical coastal site, with a partially rocky-sandy bottom with patches of *Cymodocea nodosa* meadows, and is less influenced by freshwater inputs. During the first 10 years of the study period, the Sovlja site was subjected only to minor impacts. However, over the past decade, due to tourism development, the Sovlja site has been under strong construction pressures, with the building of a marina, shipyard, tourism housing and gravel beach nourishment at the site.

A generally negative pattern was observed for all analysed diversity indices. However, certain fish species occurring only once or with a single individual in the early period were also present in the late period. This is likely why the difference between periods at the Sovlja site were not significant. In addition, the most abundant species in the early period, such as *L. mormyrus* or *Mullus surmuletus*, both very ubiquitous and abundant fish species, disappeared later in the study. Since both of these species are of high commercial interest along the coast, their abundance can be related with environmental and anthropogenic pressures^13,47,48^. The overexploitation of piscivorous predators and potential habitat modification as potential reasons for the change in abundance of sparids, *S. salpa* and *L. mormyrus*, in the surf-zone fish community were discussed^49^. The abundance of *L. mormyrus* can be further exacerbated by parasitism (*Ceratothoa italica*), particularly under environmental conditions with significant fishing pressure^50^. *Mullus surmuletus* (and *Symphodus cinereus)* recruit mainly in *Posidonia oceanica* beds^51^, while other species (*Serranus cabrilla*, *Coris julis*, *Symphodus ocellatus*, S*. rostratus*) are abundant in both seagrass beds and on rocky substrates, suggesting that the devastation of *P. oceanica* beds would severely affect *M. surmuletus* recruitment. However, in the present study, both sites are overgrown with *Cymodocea nodosa* and we witnessed its meadows reduction during study period. A sudden and drastic decline of the *C. nodosa* meadow occur due disturbances of environmental conditions, particularly those compromising the light availability^35^.

### Fishing impact

The effects of conventional fisheries management based exclusively on applying gear-specific regulations on juvenile communities, highlighting the strong potential of fishing to modify these communities were showed^47^. It is well known that intensive fishing activities in the Mediterranean basin induce higher levels of community stress^2^. It was found that more than 64.7% of juvenile species forming coastal communities are among the main targets for commercial fisheries in the southern Adriatic^52^. However, fishing practice appears to not be the dominant trigger to the observed changes in community composition in this study, as seen by the number of non-commercial fishes (NC) and BEN that were significant between periods, while high-commercial (HC) and top predator species from the categories MECA1 and MECA 2 were present without significant change in their abundance. The category of NC and BEN include species of low commercial value, mainly small benthic species such as those from the families Gobidae, Blennidae, Callionimidae, Sygnathidae together with Labridae, all of which are resident, marine species. Thus, it can be proposed that this change was more likely driven by disturbances or parameters related to hydrographic and bottom changes rather than fishing. Also, food supply is one of the possible causes than can be related with abundance decline over the years. However, generally, these species feed on a wide variety of prey items^53^ which means that food supply should not be the main driver of change.

### Climate change

Beside fishing effects, novel communities of juvenile fish in coastal areas due to the spatio-temporal rearrangement of species have been suggested to be a likely result of climate change in regions where climate has substantially altered the physical environment (see^36^ and references therein). Climate change acts on a much wider scale, having the potential to alter fish species distribution range, abundance and consequently the structure of ichthyocommunities^15,16^. The Adriatic Sea has long been recognized as a sea sensitive to climate change. Evidence of connections between the shifts in the middle Adriatic ecosystem and the northern hemisphere climate via changes in regional atmospheric conditions highlight the importance of these climate changes on the physical and biological regimes of the Adriatic Sea^54^. Accordingly, it is important to investigate temporal changes in littoral juvenile fish assemblages through their responses to environmental factors, such as sea water temperature and salinity fluctuations as important causal factors of habitat modifications due climate changes, as well as anthropogenic factors like fisheries effort. However, predictions concerning biological responses to climate change are based primarily on environmental tolerances of individual species^41^. A similar temporal pattern were found at several shallow coves within the Kornati Archipelago (Adriatic Sea), with the prevalence of five dominant species, and authors concluded that only a low amount of variation in abundance could be explained by temperature and salinity^55^, as also confirmed in the present study.

## Conclusions

Conducted multivariate analysis clearly confirmed the different composition of juvenile fish fauna between the early and late period at both sites. There was no similar pattern between sites in the early period, which is not surprising since the sites are geographically separate and under different ecological conditions^32–34^. The analysis suggested that species composition in the late period at both sites is governed by the same pattern. Studying long-term interannual changes in abundance, biomass, diversity and structure of littoral fish assemblages^48^, reported that the factors affecting littoral fish assemblages are not local but regional in nature. Cyclicity also indicates that threat communities are similar along the coast and not specific to a certain area.

Overall, this study is the first attempt to provide a new explanation of the observed negative changes in juvenile fish community compositions. The usually culprits that alter habitats are fishing pressure and climate change^18,29^. However, although environmental, fishing and trophic level indices were applied in this study to determine the factors most responsible for changes in juvenile fish assemblages in coastal nursery areas, our results did not highlight any of these factors as a potential cause. On the contrary, they suggested that something else likely lies behind these changes, such as factors related more to habitat modification, since most of the impacted species in the present study are highly sensitive to bottom degradation. In examining the sampling sites over the last 20 years, both sites have changed markedly in terms of laying concrete along the shoreline, laying moorings for boats, and gravel beaches nourishment to make beaches for swimmers^46^. At the global scale, and certainly along the Mediterranean coasts^18^, urbanization has resulted in substantial proportions of heavily modified coasts with embankments, marinas, breakwaters and seawalls. There is a broad consensus that coastal defence structures are poor surrogates for the natural habitats they replace^56^. Without a doubt, fish communities may respond to alterations to benthic substrates (see^13^ and papers therein). On the other side, despite their detrimental impact, these coastal defence constructions could play a role in the functioning of coastal ecosystems enabling highest densities of juvenile fishes on such structures^21^. As an example, mentioned authors found that *Diplodus sargus* very efficiently use such anthropogenic structures as potential juvenile habitat. But, according to our results, more negative effects can be observed from embankment and gravel beaches nourishment than positive ones due construction of piers or jetties. Moreover, the general public is unaware that these activities that serve tourism in coastal areas can have far-reaching effects on coastal communities, fisheries and climate change. Durrieu de Madron et al.^18^ highlighted their concern how the Mediterranean biodiversity will react to this changing environment, since the Mediterranean Sea is unique and evolves rapidly, with large inter-annual to decadal variability and abrupt fluctuations. A better understanding of both the bottom-up and top-down controls in marine communities will certainly require modelling efforts at both the ecosystem level, to couple low and high trophic levels, and at the species biology level, with the addition to biophysical coupling, including responses to intrinsic and extrinsic factors. For instance, how a species responds to such parameters is key information for simulating and predicting presence and abundance, and consequently determining the “healthy” composition and structure of communities.

## Material and methods

### Sampling procedure

**Figure 8:**
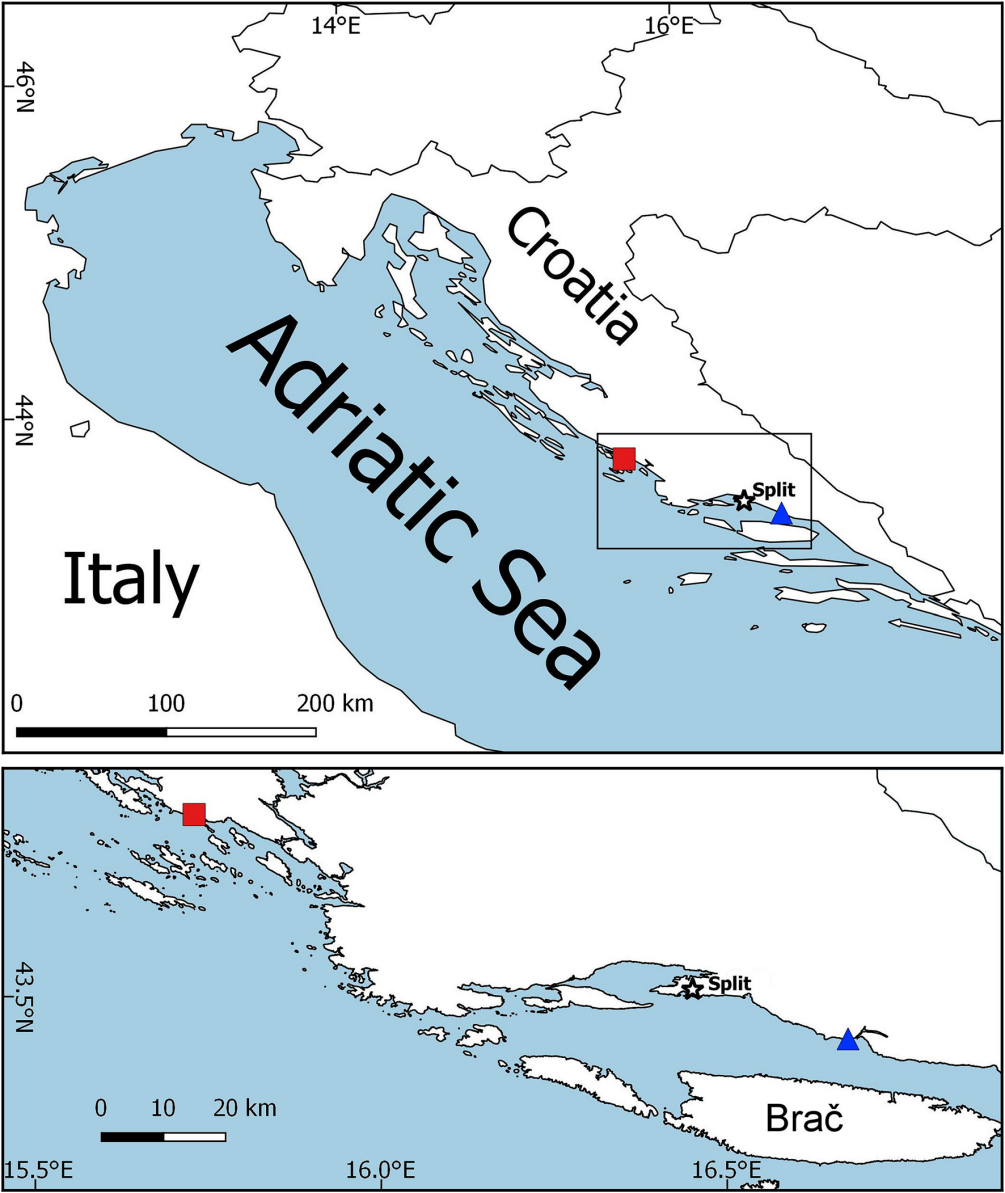
Sampling area along the eastern Adriatic coast with selected sites: (**A**) Duće (squares) and (**B**) Sovlja (circles). Map in this figure was created using software QGIS Desktop 2.18.27 (version Las Palmas) (https://qgis.org/en/site/). We generated it by our self for this manuscript using open source layers for world and Adriatic coast.

Sampling on a monthly basis (12 months) was carried out in the eastern Adriatic in 2000–2001 and 2017–2018 at two sites: Duće site (estuary of the Cetina River near Split) and Sovlja site (near Šibenik) (Fig. 8). In both years, monthly sampling of juvenile fish with three replicates (sites randomly selected separated by 10 s of meters) was conducted using a special constructed small shore seine (L = 25 m; minimum mesh size 4 mm). The seine was made of the same construction and technical features in both years, and used in the same manner to ensure comparability. Hauls were also performed in the same bathymetric range, from 0 to 2.2 m depth. Both sites were selected over a similar biotope characterized mainly by sandy or mixed substrata covered by photophilic algae alternating with patches of *Cymodocea nodosa* seagrass beds. They are of similar sizes, Duće site is 2642 m^2^ and Sovlja site is 2579 m^2^. However, Sovlja site is less influenced by freshwater inputs since it is located more than 10 km from Krka River Estuary. Mean temperature over the year in the early and late period for Duće was from 12.5 °C in January to 23.8 °C in July (mean annual 18.73 °C) and from 12.4 °C in January to 24.5 °C in August (mean annual 18.19 °C), respectively while salinity ranged from 25.4 ppt (April) to 35.3 ppt (July) and from 23.9 ppt (March) to 38.8 ppt (September) in the early and late period, respectively. Mean temperature over the year in the early and late period for Sovlja was from 12.4 °C in January to 28.8 °C in July (mean annual 18.59 °C) and from 12.7 °C in February to 25.7 °C in August (mean annual 18.15 °C), respectively while salinity ranged from 28.1 ppt (March) to 38.3 ppt (October) and from 34.7 ppt (March) to 39.8 ppt (January) in the early and late period, respectively. Continuous pier construction together with gravel beaches nourishment at Sovlja site were experienced over the study period.

In total, 36 samples (hauls) were taken per site in each period. At the time of sampling, site environmental characteristics were also determined: temperature (°C) and salinity (‰) (using a multi-parameter probe). All fish individuals were identified to the species level according to^53^, measured (total length, to the nearest 0.1 cm) and weighed (total body weight, to the nearest 0.1 g). The ethics committee of Institute of Oceanography and Fisheries approved the fish sampling that were performed in accordance with relevant named guidelines and Croatian fisheries regulations.

### Data analysis

The following indices were calculated from the species abundance data caught at each site in each period. Firstly, the number of individuals (N) and number of species (S) and mean number of individuals and species per haul were counted, together with the corresponding rank in each period and site. All species caught over the study were characterised as either high (HC), medium (MC) or low to non-commercial (NC) species depending on their market price^47^. Ecological preferences were assessed as MICA (microcarnivorous), MACA (macrocarnivorous like Scorpaenidae, Serranidae, Carangidae, Pomatomidae, Scombridae, Sphyraenidae), MECA 1 (mezocarnivorous like Labridae), MECA 2 (other mezocarnivorous like Sparidae, Mullidae), OMN (omnivorous like Mugilidae), PLA (planktivorous like Atherenidae), HER (herbivorous), BEN (benthic mezocarnivorous like Blennidae, Gobiidae, Tripterygiidae) , defined on the basis of the prevailing feeding habits and spatial organisation in the water column^13,57^. Diversity indices were also calculated for all hauls: total number of species, Margalef’s species richness (d), Pielou’s evenness (J), Shannon–Wiener diversity (SW), Simpson’s indices (D; 1-ƛ) and average taxonomic, distinctness measures in quantitative form (Δ*).

Univariate PERMANOVA was used to test the difference of all calculated indices between periods and sites. Multivariate PERMANOVA was used to test the difference of site or period effects on fish composition. Statistical analysis was performed using PRIMER (V. 7.0.13; Auckland, NZ) and graphs were prepared using SigmaPlot (v. 14.0; Systat Software Inc, San Jose, CA, USA: https://systatsoftware.com/products/sigmaplot/).

Multivariate statistical testing of the abundance data for juvenile fish assemblages used a three-factor design (Site/Period/Months), with two periods: early (2000) and late (2017). All factors were considered fixed. The additional factors Time.month (1–12 Early; 13–24 Late) and PeriodSite were combined. The data sets for each site were opened in the PRIMER workspace and combined into a single matrix (“combine DU-SO final”). Variability in the numbers of individual species in the samples was used to carry out dispersion weighting for each species^58^. Dividing counts for each species by its mean index of dispersion then ensures all species have an equivalent variability structure, and prevents the analyses from being dominated by strong outliers. After performing the Shade plot, the species *Atherina* sp. and *Sardina pilchardus* were excluded, since *Atherina* sp were the most dominant and most common species in all samples, and the majority of specimens were adults, while *Sardina pilchardus* was caught just once in high abundance, thus skewing the results.

The dispersion-weighted data was transformed by a square-root transformation, as demonstrated by^59^ for fish communities, particularly those in estuaries and nearshore coastal waters where the prevalence of juvenile and small schooling species is high. Bray–Curtis similarity was then calculated on the dispersion weighted, transformed data, using the PERMANOVA routine in the PRIMER v7 package^60–62^.

Metric Multidimensional Scaling (mMDS) ordination then used data at this same level of averaging over samples for each Site, Period, Month combination to visualise the extents to which ichthyofaunal composition differed across PeriodSite. All of these plots were constructed by calculating the distances between each pair of group centroids, i.e. the relevant average in the ‘Bray–Curtis space’ of all samples^61^.

The RELATE statistic (p) was employed to test whether the pattern of temporal catch composition change conforms to cyclicity. If there is no tendency to cyclicity, then p will be close to zero^63^. In order to identify the main environmental variable driving temporal change, the BEST routine was applied. Separate two-way crossed analysis of similarities ANOSIM^59^ was used to interpret the relative size of the overall Period and Site effects on fish compositions, using the same resemblances as for the PERMANOVA tests. The species mainly contributing to the separation between the factor PeriodSite were determined using the SIMPER (similarity percentages) procedure that indicates the average contribution of each species to the dissimilarity between groups of samples.

To explore things further, we used the CAP analysis (Canonical Analysis of Principal coordinates). This was initially run separately for each of the two factors: “Site” and “Period” and then merged into a scatter plot. Particularly, CAP was used to estimate the accuracy of fish composition and environmental variables (temperature and salinity).

Segmented bubbles of proportional sizes, representing the dispersion-weighted and square-root transformed abundances of selected families (Gobiidae and Blenidae), fishing category (NC and HC) and trophic value (BEN and MECA 2) were overlaid on the corresponding mMDS plot. This illustrates the abundance trends exhibited by some of the indices that varied conspicuously between periods and/or sites, and thus how they contribute to the structure of this plot.
